# Very early migration of a calcar-guided short stem: a randomized study of early mobilization and the influence of a calcium phosphate coating with 60 patients

**DOI:** 10.1038/s41598-023-50829-3

**Published:** 2024-02-15

**Authors:** Stefan Budde, Alexander Derksen, Christof Hurschler, Peter Fennema, Henning Windhagen, Jochen Plagge, Thilo Flörkemeier, Gabriela von Lewinski, Yvonne Noll, Michael Schwarze

**Affiliations:** 1 Hannover Medical School, Department of Orthopaedic Surgery, Diakovere Annastift, Anna Von Borries Str. 1-6, 30625 Hannover, Germany; 2 Evangelisches Klinikum Bethel, Universitätsklinikum OWL der Universität Bielefeld, Burgsteig 13, 33617 Bielefeld, Germany; 3 AMR Advanced Medical Research, Männedorf, Switzerland; 4 Department for Medical Technology, Hochschule Bremerhaven, An Der Karlstraße 8, 27568 Bremerhaven, Germany

**Keywords:** Randomized controlled trials, Musculoskeletal system, Orthopaedics

## Abstract

This study analyzed the migration of a calcar-guided short stem to determine the course of very early migration, as well as evaluated the effect of an additional calcium phosphate (CP) coating on a titanium plasma spray (TPS) coating, which has not been analyzed previously. Sixty patients were enrolled in this study and were treated with the A2 calcar-guided short stem. The implant coating was randomized with either the TPS or an additional CP coating, and radiostereometric analysis was performed with the baseline measurement before initial weight-bearing, along with follow-up examinations at 1 week, 6 weeks, 3 months, and 6 months. Implant migrations were 0.27 mm (standard deviation [SD], 0.13 mm) and 0.74 mm (SD, 1.11 mm) at 1 week and 6 months post-surgery, respectively, and 65% and 87% of the implants reached their final position 1 week and 6 weeks after surgery, respectively. After 6 weeks, 3 months, and 6 months, a significant increase was noted in the migration of the CP coating group vs. that of the TPS coating group. Upon the final observation at 6 months, the groups displayed on average a 0.74-mm migration. Most of the analyzed implants ceased migration within the first week post-surgery, but the CP coating demonstrated a higher and more prolonged migration compared to the TPS coating.

## Introduction

Previous studies on short stems have revealed a distinct pattern of migration in cementless short-stem total hip arthroplasty (THA) when analyzed using radiostereometric analysis (RSA). For instance, several individual prosthetics demonstrated an initial migration of approximately 1 mm between the postoperative baseline examination and the first follow-up at 3 months^1–8^. The RSA-ISO standard 16,087:2013 recommends a reference examination “within 5 days, preferably before weight-bearing.” Thus, if full weight-bearing is allowed on the first postoperative day and if the reference examination is not scheduled before the patient reinitiates mobilization, the earliest phase of migration will remain undetected.

The migration patterns of short hip stems may be influenced by the implant coating, as previously demonstrated among conventional implants^9^. Since short-stem implants have a smaller contact surface between the implant and the bone compared to conventional stems, in the earlier years of their development it was presumed that they may be at a higher risk of excessive implant migration and loosening, This was also due to the fact that, at that time, no clinical or registry data were available for these devices^10^. In response, short-stem manufacturers have made every effort to improve osseointegration, so most implants are only available with an additional calcium phosphate (CP) coating to facilitate bone ongrowth^11^. The CP coating is meant to be resorbed during the first 3 months and has shown increased implant osseointegration compared to hydroxyapatite coatings^12^. However, the effect of the additional coating on the migration of short stems has not been analyzed.

This study aimed to quantify the effects of initial weight-bearing within the first week post-surgery and to determine the exact course of the very early migration of a calcar-guided short stem by adding two additional RSA follow-up examinations within the first 3 months to the standard protocol. The primary hypothesis is that the mean total migration of a calcar-guided short-stem implant with an additional CP coating (Bonit®, DOT, Rostock, Germany) is less than that of an implant with a bare titanium plasma spray (TPS) coating within the first 6 months post-surgery.

## Materials and methods

### Clinical study design

Ninety-eight patients scheduled for THA were examined for participation in this study. After applying the inclusion and exclusion criteria (Table 1), 60 (38 female and 22 male) patients were enrolled in this study (Table 2). All patients provided informed consent to participate in the study.

**Table 1:** Inclusion and exclusion criteria.

Inclusion criteria	Exclusion criteria
• Require primary total hip replacement for radiographically proven primary coxarthrosis, dysplasia coxarthrosis, or femoral head necrosis without affection of the femoral neck • Willing to participate in the trial and intend to be available for the scheduled follow-up visits • Normal motor function of the lower extremities, documented by clinical examination • Age on day of surgery: 30–70 years	• Previous bony or soft tissue surgery on the affected proximal femur (arthroscopic surgery was excluded) • Infection of the affected hip joint or systemic infection • Known osteoporosis diagnosed by a healthcare professional • CCD angle above 145 • CCD angle below 120 • Diseases of the cardiovascular system that result in a highly reduced ability to exercise in everyday life (e.g., ASA score 3 or 4) • Proven allergy to components of the implanted prosthesis • Known neurological diseases, with alterations in motor function • Pregnant or lactating women • BMI over 30 • Known alcoholism or other relevant addictive disorders
Subsequent exclusion from the clinical trial:	
• Occurrence of fracture of the proximal femur or acetabulum • Intraoperative circumstances necessitate implantation of a prosthesis type other than that assessed in the study • Revision surgery due to prosthesis infection, loosening, or fracture during the clinical trial. A single soft tissue infection within the first 6 weeks of implantation did not lead to exclusion • The subject revokes informed consent • Less than four RSA markers are clearly visible in the post-operative examination • The RSA condition number in the post-operative examination is greater than 120 A revision rate exceeding the benchmark of 10% per year was defined as a criterion triggering premature abortion of the study

**Table 2:** Baseline demographics and clinical characteristics.

	TPS	CP	Total
Number of patients	30	30	60
Age	55.9 (SD, 8.7) years (30.0–70.0 years)	59.7 (SD, 8.0) years (30.0–70.0 years)	57.8 (SD, 8.5) years (30.0–70.0 years)
Sex (Male/Female)	9/21	13/17	22/38
Body mass index	24.5 (SD, 3.3) kg/m^2^ (19.3–29.7 kg/m^2^)	25.3 (SD, 2.5) kg/m^2^ (20–29 kg/m^2^)	24.9 (SD, 2.8) kg/m^2^ (19.3–29.7 kg/m^2^)
Stem geometric body (shape G/shape B)	8/22	5/25	13/47

*TPS* titanium plasma spray, *CP* calcium phosphate, *SD* standard deviation.

All patients were treated using an A2 calcar-guided short-stem femoral component (Artiqo GmbH, Lüdinghausen, Germany). The A2 stem is available with two different geometric bodies: shape B for varus and neutral anatomies of the proximal femur and shape G for a valgus anatomy. The geometric shape was selected by the surgeon based on preoperative templating and intraoperative visual and fluoroscopic impressions. In addition, the stem was coated with either TPS (referred to as TPS) or TPS with an additional CP coating (referred to as CP). The study was conducted at the university hospital at Hanover Medical School, Orthopaedic Department, in Diakovere Annastift.

The patients were allocated to each group by generating a randomized blocked list (block size 4–8, generated with the statistical program R and the package blockrand^13,14^. The list was unavailable to the treating physician and was kept in 60 sealed numbered envelopes by the clinical trial management personnel.

### Surgical protocol

**Figure 1:**
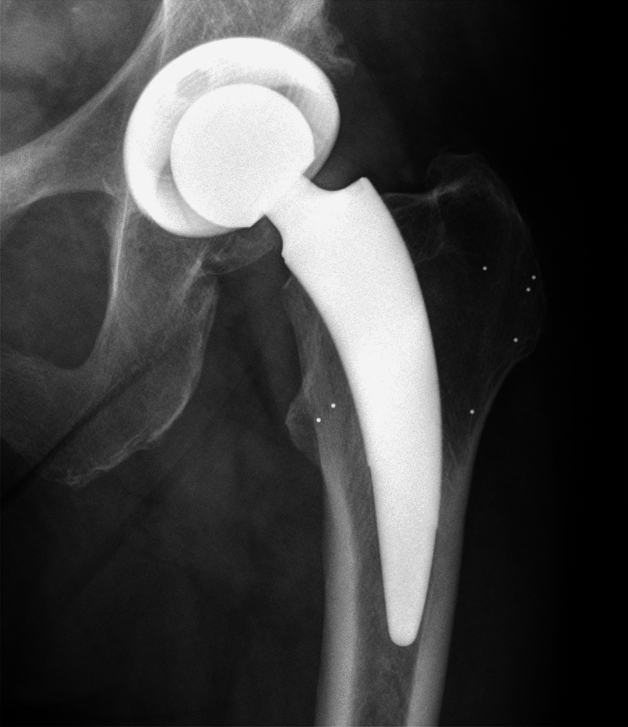
X-ray image of the implant and the surrounding tantalum markers in the trochanter major and minor.

Surgical treatment was performed according to routine procedures, by one of the three senior surgeons (SB, GvL, TF) experienced in short-stem THA, via an anterolateral approach at one university hospital center, where all data were collected. For subsequent RSA analyses, 6–10 tantalum marker beads (Tilly Medical Products AB, Sweden [diameter = 1.0 mm]) were inserted into the greater and lesser trochanters using a cannula (Fig. 1).

### RSA analysis

Baseline RSA images were obtained either on the day of surgery or the following morning in cases of late surgery. In all patients, initial weight-bearing was allowed under supervision on the first postoperative day and after the RSA baseline examination. RSA follow-up examinations were performed on the seventh postoperative day in the inpatient setting, after 6 weeks, and after 3 and 6 months. To perform RSA radiography, the subject was laid on the X-ray table in the supine position, and two radiographs were simultaneously taken using two different X-ray tubes, which allowed for the software-based (model-based RSA version 4.11, RSAcore, Leiden, the Netherlands) evaluation of implant migration. The implant surface models required for the model-based RSA were obtained from the implant manufacturer, and to avoid possible errors resulting from the surface coating, additional surface scans of the implant samples were generated by optical scanning. In addition, surface deviations from the computer-aided design (CAD) data were evaluated three-dimensionally, where the surface deviation among the scanned implants compared with the CAD models was 0.02 mm on average, with a maximum deviation at single locations of + 0.15 mm across the surface coating area.

The coordinate system of the calibration box was defined as the reference for the measurements, where the positive x-, y-, and z-directions correspond to the medial, cranial/proximal, and anterior directions, respectively. Migration was defined as the magnitude of the resultant movement vector, which is (Tx^2^ + Ty^2^ + Tz^2^)^0.5^, of the geometric center of the implant. Meanwhile, the RSA parameters and procedures (Appendix Table A.1) were defined according to standard guidelines (ISO 16087:2013).

The patients and the radiological technical assistants performing the RSA measurements were blinded, as were those performing software-based RSA.

For the analysis of whether an implant is still migrating or has reached a fixed position, a threshold of 0.3 mm was selected, which was slightly above the precision of the RSA setup used, as determined by a double examinations (precision of total migration, 0.27 mm).

### Ethics and registration

The local ethics committee approved this study (Institutional Review Board of Hannover Medical School, No. 7663MPG-LKP Mono, October 2018), and it was registered in the German Clinical Trials Register (DRKS00017645) on 29/07/2019. All methods were carried out in accordance with relevant guidelines and regulations (ISO 14155, MDR Article 77(5), EU guideline EU-2023/C163/06, and the Declaration of Helsinki).

### Statistical analyses

A statistical evaluation was performed using linear mixed model analysis in R with the lmertest package^13,15^, and the model was fitted using restricted maximum likelihood estimation. Patient ID was treated as a random effect, and the factors of time and implant coating and their interactions were treated as fixed effects. A secondary analysis was performed with patient ID as a random effect and time, implant shape, and their interactions as fixed effects. This approach was selected to account for repeated measurements of individual subjects and for partially missing observations, and a p value < 0.05 was considered statistically significant.

The total sample size of 60 patients was based on the primary endpoint of the full 2-year study, which was calculated to show the superiority of the study group’s 30% lesser migration than the historical control group, which had a migration of 0.86 (standard deviation [SD], 0.97) mm. The comparison was planned for log-transformed data with a power of 80% and a two-sided significance level of 5%, utilizing a t-test.

## Results

The patients were recruited between January 2019 and December 2020, and the study ended in November 2022 according to the protocol. Patient flow is detailed in Table 3.

**Table 3:**
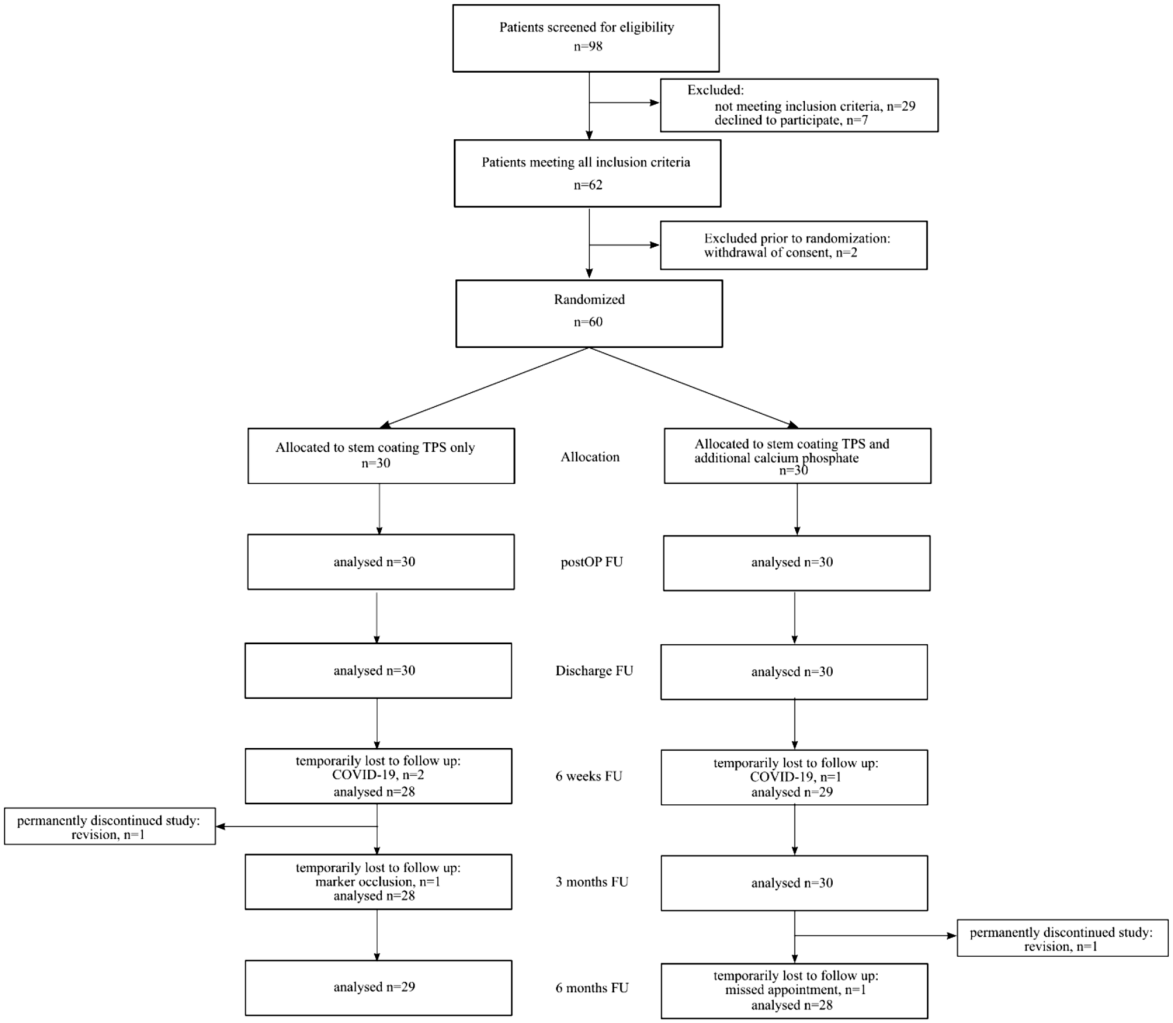
Participant flow diagram.

Two patients underwent revision surgery due to aseptic loosening, in the first of whom the implant was undersized and lacked contact with the distal cortex of the femur, leading to substantial migration, as detected in the follow-up examination 6 weeks post-surgery. Meanwhile, the second patient showed substantial migration after 6 weeks, in addition to a visible decline in calcar bone density, though it was clinically unobtrusive. After further migration at 3 months, in conjunction with a fall on a slippery snow surface, revision was indicated. None of the patients was included in the statistical evaluation of migration over time, as they are not representative of the behavior of the coatings.

**Figure 2:**
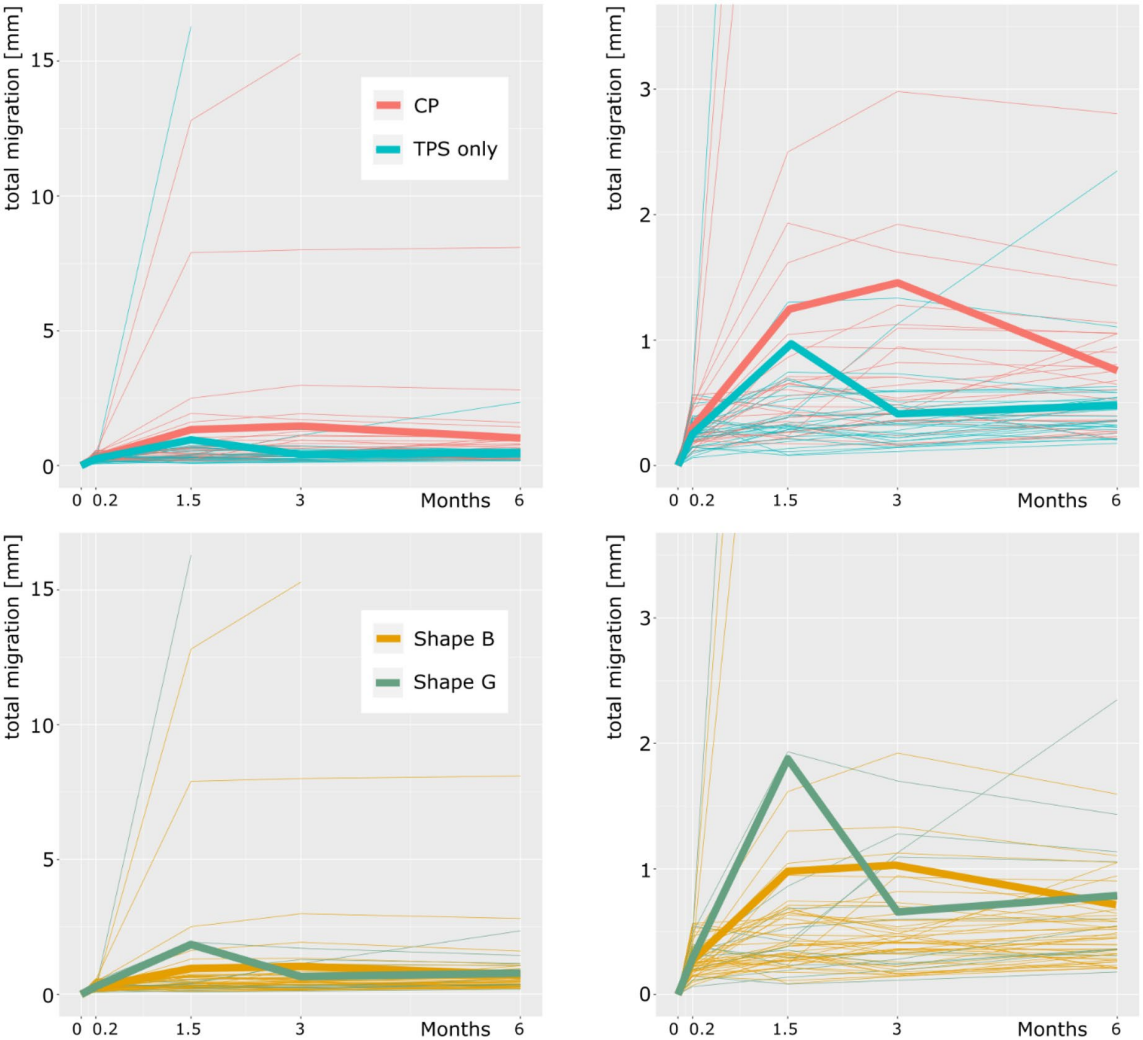
Total migration in the first 6 months after baseline. Left: Migration of all patients. Right: Migration of all patients: scale reduced range to 3.5 mm for better visualization of early migration. Top: Migration according to the type of coating (red: calcium phosphate, blue: titanium plasma spray). Bottom: Migration according to the shape of the prosthesis (yellow: type B, green: type G).

Seven days post-surgery, the implants migrated 0.27 mm (SD, 0.13) on average, with the CP and TPS coating groups having displayed 0.29-mm (SD, 0.14) and 0.25-mm (SD, 0.12) migrations, respectively (p = 0.852, Fig. 2).

After 6 weeks, the implants migrated 1.15 mm (SD, 2.81) on average, with the CP and TPS coating groups having displayed 1.33-mm (SD, 2.64) and 0.96-mm (SD, 3.01) migrations, respectively (p = 0.019). Of the 57 patients available for follow-up at 6 weeks, 17 displayed a migration of > 0.3 mm when compared with the discharge analysis. Among these patients, 11 had CP and six had TPS coatings, whereas four patients demonstrate a G shape and 13 a B shape.

After 3 months, the implants migrated on average 0.96 mm (SD, 2.21). The CP and TPS coating groups displayed 1.46-mm (SD, 2.99) and 0.41-mm (SD, 0.28) migrations, respectively (p = 0.009, Fig. 2). Of the 58 patients available for follow-up at 3 months, 7 displayed a migration > 0.3 mm relative to the 6-week analysis. Of these patients, six had a CP coating and one a TPS coating, whereas three patients had a G shape and four a B shape.

After 6 months, the implants migrated 0.74 mm (SD, 1.11) on average, with the CP and TPS coating groups having displayed 1.00-mm (SD, 1.49) and 0.48-mm (SD, 0.41) migrations, respectively (p = 0.019, Fig. 2, Table 4). The most prominent direction of migration was a subsidence along the y-axis of 0.33 mm (SD, 2.17). There was no statistically significant difference in migration between the two stem shapes (p = 0.816). Of the 57 patients available for follow-up at 6 months, two displayed a migration of > 0.3 mm relative to the 3-month analysis. Among these patients, one had a CP coating, and one had a TPS coating. Furthermore, one patient had a G shape and one a B shape.

**Table 4:** Total migration after 6 months (by implant shape and implant coating).

Total migration after 6 months	Shape B (n = 46)	Shape G (n = 11)	Shape B + Shape G (n = 57)
TPS (n = 29)	0.42 mm, SD: 0.20 mm (n = 22)	0.67 mm, SD: 0.75 mm (n = 7)	0.48 mm, SD: 0.41 mm (n = 29)
CP (n = 28)	1.01 mm, SD: 1.61 mm (n = 24)	1.00 mm, SD: 0.45 mm (n = 4)	1.00 mm, SD: 1.49 mm (n = 28)
Combined (n = 57)	0.73 mm, SD: 1.19 mm (n = 46)	0.79 mm, SD: 0.65 mm (n = 11)	0.74 mm, SD: 1.11 mm (n = 57)

Statistical analysis: p = 0.019 comparing TPS vs. CP coating and p = 0.816 comparing shape B vs. shape G.

*TPS* titanium plasma spray, *CP* calcium phosphate, *SD* standard deviation.

**Figure 3:**
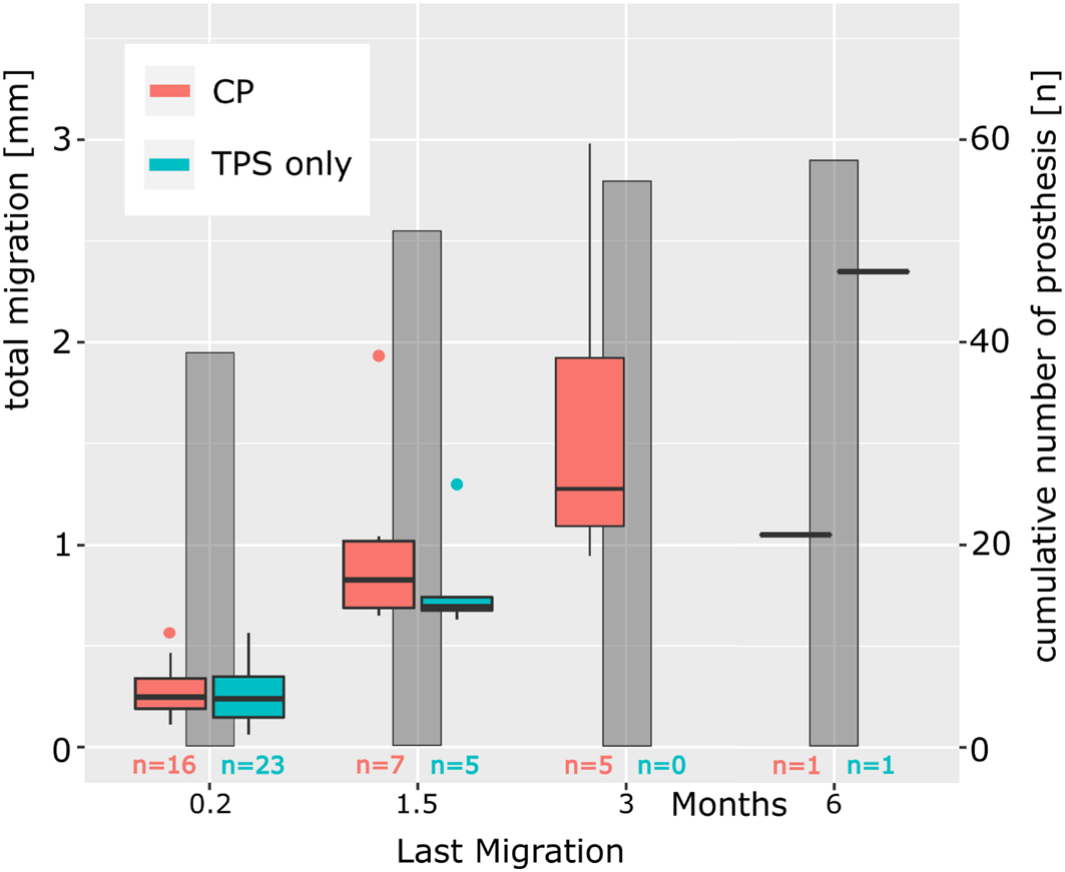
Total migration at the time point at which migration was detected for the last time for calcium phosphate (CP) and titanium plasma spray (TPS) only. Cumulative number of prostheses having ceased migration, with respect to a 0.3-mm threshold (gray bars). The revised implants (n = 2) have been excluded.

One week after surgery, 65% (39/60) of the implants reached their final position, so further migration was defined by a threshold of 0.3 mm (Fig. 3), and 6 weeks after surgery, 88% (51/58) of the implants reached their final position within this threshold. Migration continued beyond the 3-month follow-up in only 3% (2/57) of patients, and the implants that still migrated at 6 weeks displayed a larger than average migration compared with all implants (Fig. 3).

The implants shown at 0.2 months did not migrate > 0.3 mm after this follow-up examination. At 1.5 months, seven implants with an additional CP coating showed a > 0.3-mm migration compared with the 0.2-month follow-up, but migration ceased at this time point, with a median total migration of 0.8 mm (compared with the baseline measurement immediately after surgery).

## Discussion

In this study, we investigated the migration of a short-stem implant with two different coatings. Contrary to our expectations, we observed increased migration of implants with an additional CP coating compared with those having a TPS-only coating.

Several studies have analyzed the migration behavior of cementless total hip implants, and detectable initial settling of the implants within the first 3 months is often observed^3,5,8,16^. After 3 months, the implants usually stabilize. The shape of the migration graph corresponds to migration pattern B, which, according to Krismer et al., indicates good osseointegration^17^. This benign migration pattern suggests favorable long-term results, whereas the absolute value of the initial implant migration within 3 months provides information about the primary stability of the implants.

For an adequate interpretation of the primary stability of the implants observed in this study, there must be comparisons with other implants in the same group. When identifying implants in the same group, conventional and short stems cannot be plainly differentiated because the mere length of an implant does not sufficiently reflect its biomechanical behavior. Several different short-stem classifications have been suggested^18–20^, and the most appropriate categorization of the A2 stems analyzed in this study was as calcar-guided (partially) femoral neck-preserving short stems. Of the several studies that have examined cementless hip implants, only a few have focused on comparable implants in this group, namely, the Metha (Aesculap AG, Tuttlingen, Germany)^21^, Nanos (Smith & Nephew plc, London, UK)^5^, and Optimys (Mathys AG, Bettlach, Switzerland)^22^ stems. All these stems aim, at least partially, for proximal load transfer into the femoral bone with metaphyseal anchorage.

Analysis of RSA data can be performed using different outcome parameters. Although the present study analyzes the resulting total migration, other studies describe the maximum total point motion or focus on the translational and rotational movements along or around three different axes^3,16,22^. In contrast, the highest proportion of total migration can usually be attributed to the subsidence of the stem, which is the distal migration along the y-axis. This parameter is available for all the aforementioned studies. When comparing the average subsidence of the different implants in the cited studies after 6 months, the Metha stem showed substantially more migration, with 0.83-mm subsidence, compared with the Optimys (0.21 mm) and Nanos (0.20 mm) stems. Moreover, the A2 stem showed an overall subsidence of 0.33 mm after 6 months; thus, it belongs to the implants within this group with a rather high primary stability.

The actual fixation stability depends on the implant, patient’s anatomy, implantation procedure, and resection height of the femoral neck. For the A2 stem, two different fixation principles can be observed: whether the femoral neck resection level is high (at the height of its smallest diameter) or low (at its base). In the first scenario, a “diagonal anchorage” can be assumed, with a major proportion of the load transferred to the closed cortical ring of the femoral neck and a second point of support at the distal tip of the implant. In the second scenario, a “fit and fill” principle of load transfer occurs, with the implant often having bicortical contact in the diaphysis and, thus, with a major proportion of the load being transferred to the meta-diaphyseal transition zone and into the proximal diaphysis. In addition, a small intramedullary canal found in type A configurations of the proximal femur, according to the Dorr classification^23^, may lead to bicortical contact of the implant in the diaphysis and thus to a more distal load transfer. A sharp line between diagonal anchorage and the fit and fill technique cannot be drawn because the transition between these theoretical principles is fluent and, in practice, most cases display a mixture of both. Therefore, an attempt to distinguish between them was not considered helpful in this study.

A special feature of the A2 stem is that it has two different implant body geometries, suggesting that it may be better treated as two completely different implants for RSA analysis. However, a separate analysis of the two geometries (shapes B vs. G) did not reveal significant differences (p = 0.705) in terms of implant migration. Thus, we chose not to differentiate between the two geometries when further discussing their migration.

In most previous RSA studies, the protocol required the first follow-up examination after only 3 months, and the duration of initial migration is commonly considered 3 months. Furthermore, baseline (reference) images are often obtained after initial load-bearing and, in some cases, not until 6 weeks postoperatively^24^. To date, no explicit data have been reported concerning what exactly happens within the first 3 months post-surgery and, particularly, not with respect to the baseline data, obtained before initial mobilization (i.e., pre-load bearing). Therefore, a particular focus of this RSA study was the distinct observation of migration within this period by performing baseline measurements before initial load-bearing and by adding additional follow-up examinations after 7 days and 6 weeks.

As the baseline RSA measurement was performed before loading and initial mobilization of each patient, the follow-up examination 1 week after surgery provided information about the effect of patients’ early mobilization. The magnitude of very early migration within the first week compared with migration after 6 months was rather low (0.27 mm vs. 0.74 mm) when comparing the mean values of total migration. However, notably, 39 of the 60 implants had already reached their final position 1 week post-surgery when defining a further migration of 0.3 mm as a threshold. Thus, the considerably higher mean value of total migration detected after 6 weeks was caused by the remaining 33% of the implants. That is, several implants showed very little migration over a short period, whereas only a few implants showed longer-lasting migration, thereby increasing the mean values of migration to a comparatively high extent.

The fact that 65% of the observed implants ceased migration 1 week post-surgery is especially interesting in consideration of the predominant understanding of osseointegration as a continuous process lasting approximately 3 months^1^. This implies that completion of osseointegration may not be a decisive factor when the early migration phase ends. Consequently, the question arises as to whether bioactive coatings, such as the CP coating tested in this study, which are intended to enhance bone ongrowth, have a positive effect on early implant migration.

The analysis of the effect of an additional CP coating on early implant migration was another important focus of this study, with data showing that the addition of a CP coating does not have a positive influence on implant migration. Interestingly, the pure TPS coating showed significantly lower early migration than the coated group. In addition, regarding the implants showing ongoing migration after 7 days, the distribution of an additional CP coating versus the TPS coating alone showed that a considerably higher number of CP-coated implants migrated for > 1 week (14 vs. 7) and for > 6 weeks (7 vs. 1).

Bioactive hydroxyapatite (HA) coatings have demonstrated positive effects on the osseointegration of implants in animal studies^6,25^. However, there is no evidence that this effect has any clinical benefit for improving patient outcomes or implant survival. In this respect, RSA studies are of significant interest because they allow the prediction of aseptic loosening within certain limits. Only one RSA study has reported the influence of an additional HA coating on implant migration by analyzing the Zweymueller stem with and without an additional HA coating. Consistent with the results of our study, the authors did not detect superiority in the additional HA coating regarding implant migration after 2 years^26^ and 5 years^27^. However, the Zweymueller stem is a conventional long stem and is a group of implants for which an additional bioactive coating is used less frequently than for short stems.

The present study is the first to analyze the effects of bioactive coatings on short stems, with the presented data suggesting that expenses for additional CP coatings may be unnecessary. Significantly reduced primary stability is observed in terms of increased and prolonged early migration, potentially explained by the stronger bonding between the CP layer and bone than the bonding between the coating and implant itself, leading to separation at the latter interface and even prolonged migration^28^. A different, similar explanation can be based on the fact that the CP coating degrades over time, so the boundary between the implant and surrounding tissue migrates, whereas it remains static in the case of the TPS coating^29^. Further, degradation of the CP layer can reduce primary stability if it occurs faster than bone growth. The third aspect concerns the rheological characteristics of the coating: the TPS coating shows a coarse macroporous surface that, to a small extent, is leveled by the additional CP coating, potentially leading to a reduction in friction.

A limitation of the present study is that no clinical outcome data were available. Furthermore, the interpretation of the presented results may be even more conclusive when considering the further course of migration of the observed implants. However, the focus of this study was on early migration patterns, and the study is still ongoing. RSA data are being collected and analyzed over a 2-year follow-up period, including clinical outcome data and conventional radiological analysis. After study completion, the data will be presented with a clinical focus that is outside the scope of the preliminary data. The presented aspects are meant to focus on basic studies, concentrating on the biomechanical aspects of the study and on novel findings regarding very early implant migration and the influence of implant coating.

## Conclusions

Most of the analyzed implants ceased to migrate within the first week after surgery, whereas a few demonstrated continuous migration increases in the mean values of migration during later follow-up examinations. Moreover, the addition of a CP coating does not have a positive effect on implant migration, as it leads to significantly greater and more prolonged migration compared with the pure TPS coating.

### Supplementary Information

Supplementary Information.

## Data Availability

The datasets generated and analyzed during the current study are not publicly available due to obligations to observe the confidentiality of patient data but are available from the corresponding author upon reasonable request.
