# Supplementary material for: Very early migration of a calcar-guided short stem: a randomized study of early mobilization and the influence of a calcium phosphate coating with 60 patients
Source: Sci Rep. 2024 Feb 15;14:3837. doi: 10.1038/s41598-023-50829-3 (PMC10869691; doi:10.1038/s41598-023-50829-3)
Supplement: Supplementary file 1 — Supplementary Information. [file 41598_2023_50829_MOESM1_ESM.docx]

**Appendix**

Table A.1: Radiostereometric analysis parameters and equipment used for image acquisition and analysis

| **Parameter** | **Value** | | |
| --- | --- | --- | --- |
| Precision by double examination on 15 patients: Translation (Tx, Ty, Tz)  Rotation (Rx, Ry, Rz) | Tx: 0.006 +/–0.101 mm  Rx: 0.003 +/–0.842° | Ty: 0.054 +/–0.144 mm  Ry: 0.107 +/–1.948° | Tz: 0.008 +/–0.392 mm  Rz: 0.042 +/–0.270° |
| Calibration cage | Carbon Box Leiden 10 Hannover | | |
| X-ray tubes | 2x SRO3310 ROT 360 (Philips) | | |
| Tube voltage/current | 90 kV/10 mAs | | |
| Angle between x-ray paths | 40° | | |
| X-ray cassette | 36 × 43 cm IP Cassette Type CC (Fuji) | | |
| Cassette digitizer | PCR Eleva Corado (Philips), resolution: 125 dpi | | |
| RSA software version | Medis Specials Model-based RSA 4.11 | | |
